# A flexible three-dimensional electrode mesh: An enabling technology for wireless brain–computer interface prostheses

**DOI:** 10.1038/micronano.2016.12

**Published:** 2016-05-23

**Authors:** Zhuolin Xiang, Jingquan Liu, Chengkuo Lee

**Affiliations:** 1 Department of Electrical and Computer Engineering, National University of Singapore, 4 Engineering Drive 3, Singapore 117583, Singapore; 2 Singapore Institute for Neurotechnology (SINAPSE), National University of Singapore, 28 Medical Drive, #05-COR, Singapore 117456, Singapore; 3 Center for Intelligent Sensors and MEMS, National University of Singapore, 4 Engineering Drive 3, Singapore 117583, Singapore; 4 NUS Suzhou Research Institute (NUSRI), Suzhou Industrial Park, Suzhou 215123, China; 5 National Key Laboratory of Science and Technology on Micro/Nano Fabrication, Department of Micro/NanoElectronics, Shanghai Jiao Tong University, Shanghai 200240, China

**Keywords:** drawing lithography, flexible electrode, neural interfaces, 3D microneedle electrode

## Abstract

The neural interface is a key component in wireless brain–computer prostheses. In this study, we demonstrate that a unique three-dimensional (3D) microneedle electrode on a flexible mesh substrate, which can be fabricated without complicated micromachining techniques, is conformal to the tissues with minimal invasiveness. Furthermore, we demonstrate that it can be applied to different functional layers in the nervous system without length limitation. The microneedle electrode is fabricated using drawing lithography technology from biocompatible materials. In this approach, the profile of a 3D microneedle electrode array is determined by the design of a two-dimensional (2D) pattern on the mask, which can be used to access different functional layers in different locations of the brain. Due to the sufficient stiffness of the electrode and the excellent flexibility of the mesh substrate, the electrode can penetrate into the tissue with its bottom layer fully conformal to the curved brain surface. Then, the exposed contact at the end of the microneedle electrode can successfully acquire neural signals from the brain.

## Introduction

Brain–computer prostheses provide a bridge for humans to understand and communicate with the nervous system. Neural interfaces are a key component enabling wireless brain–computer prostheses for long-term implantation. In the last decades, researchers have developed various types of neural interfaces based on advanced functional materials and fabrication technology^1–3^. Because a two-dimensional (2D) structure can be shaped precisely and easily, most neural interfaces are fabricated into planar geometries, which are known as neural probes. However, neural tissues are normally three-dimensional (3D) structures, and 2D neural probes are limited to recording signals from only planar brain regions^4,5^. Therefore, 3D microneedle electrodes need to be developed to acquire additional information from the nervous system^6,7^.

Typically, the fabrication of 3D microneedle electrodes can be classified into two groups: assembling 2D probe combs into 3D structures and etching bulk materials into 3D devices. In the first approach, 2D neural probes are fabricated by standard planar surface micromachining and then assembled in a specially designed platform to form a 3D probe array^8,9^. Fabrication technologies in the second approach are relatively more diverse depending on the bulk materials. For example, reactive-ion etching^10^ and anisotropic wet etching^11–13^ are normally deployed to fabricate silicon-based out-of-plane microneedle structures. Metal microneedle structures are often formed using an electroplating process onto a seeding layer that is predefined by a polymer micromold^14^. Polymer 3D microneedle structures are created using stainless steel molding technology^15^, inclined ultraviolet (UV) exposure technology^16^, polydimethylsiloxane molding technology^17,18^, and etched lens backside exposure technology^19^. Because these approaches require special equipment or platforms, microneedle structures that are fabricated either by assembling 2D probe combs or etching bulk materials are expensive and time consuming.

Another problem for 3D microneedle electrodes is the mismatch in the mechanical properties between their rigid substrate and soft biological tissues. On the basis of tissue type, age and the health of the organism, functional components *in vivo* normally have well-defined mechanical properties characterized by elastic moduli that fall within a relatively narrow range. Conventional 3D microneedle electrodes made of silicon or glass are usually many orders of magnitude stiffer than these targeted components, which hinders the device’s capability to conformally integrate with the curved surface of the target tissues. Consequently, the rigidity of the 3D electrode substrate leads to a space between the targeted tissue and the recording contacts, which may shift the electrode location during the testing process. Furthermore, to keep the electrode with a rigid substrate in the targeted position, excessive pressure needs to be applied to fasten it to the tissue. This pressure has been reported to cause serious damage to the tissues, including inflammation and nerve degeneration^20–22^.

Moreover, it is highly desirable to access different layers in the nervous system because they may have distinctive functions. For instance, selectively recruiting different layers in the peripheral nerve results in a graded force generation in various muscle groups^23–25^. Stimulation in different layers of the cerebral cortex induces various activations of neuronal subtypes and their complex connection to subcortical regions, which leads to diverse cognition, sensory perception and motor control^26^. The Utah Slanted Electrode Array (USEA) was proposed to provide comprehensive access to multiple independent motor nerves^27,28^, whereas a varying length polymer microneedle array was developed as a waveguide to induce light stimulation in different layers of rat brains. However, both techniques had limitations. The length of USEA was restricted by the bulk silicon thickness and the etching process, while for the varying polymer microneedle array, the longest microneedles were fixed in the central area due to the droplet profile. These drawbacks limited their applications.

Taking all these limitations into consideration, we demonstrate that a unique 3D microneedle electrode on a flexible substrate, which can be fabricated without complicated micromachining techniques, is conformal to tissues with minimal invasiveness; furthermore, it can be applied to different functional layers in the nervous system without length limitations. The microneedle electrodes were fabricated using drawing lithography technology and insulated using a parylene coating process. With process optimization, the length of the 3D microneedle electrode can be determined via the design of the 2D mask pattern. With the sufficient stiffness of the electrode and the excellent flexibility of the substrate, the microneedle was able to penetrate into the tissue with its bottom layer fully conformal to the curved brain surface. Then, the exposed contact at the end of the microneedle electrode was able to successfully acquire spike signals from the brain.

## Materials and methods

### Design of the flexible microneedle electrodes

**Figure 1:**
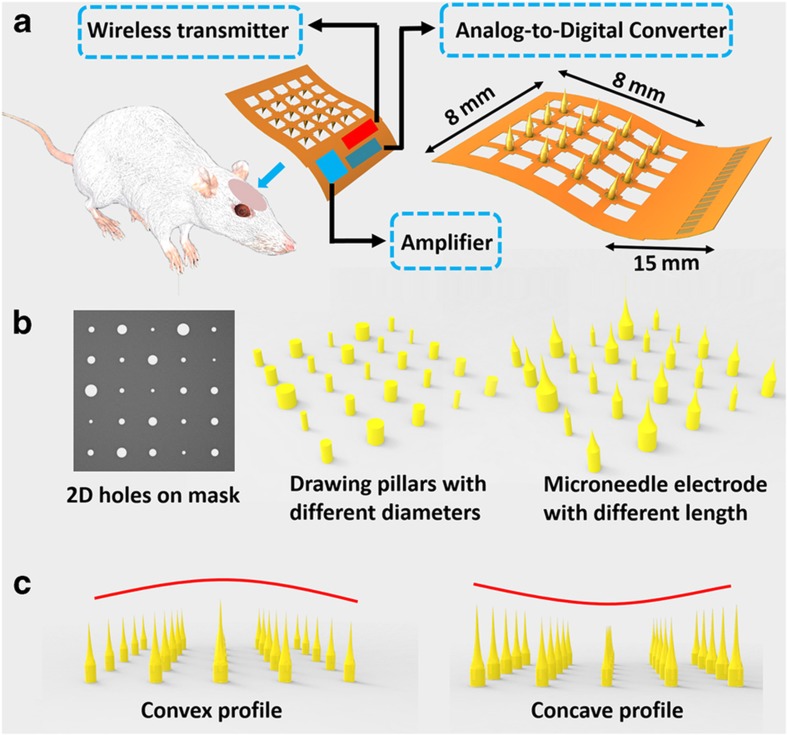
A schematic depiction of the flexible microneedle electrode. (**a**) The design and geometry of the flexible microneedle electrode. (**b**) The 2D mask design that determines the 3D microneedle electrode length. (**c**) The microneedle electrode array with different profiles. 2D, two dimensional; 3D, three dimensional.

The proposed flexible microneedle electrode is one of the key components in wireless brain–computer interface prostheses. A schematic drawing is shown in Figure 1. In the final system, all the electronic components, including the amplifier, analog to digital converter and wireless transmitter, can be integrated on the flexible substrate (Figure 1a). The microneedle electrode itself can be divided into three different sections. At the rear, a 15-mm long stripe is designed for easy handling and packaging. This is where the external contact electrodes are located. There are 16 electrode pads, each with an area of 500 μm×300 μm. These dimensions ensure an easy connection of the device to the flexible printed circuit connectors. Another advantage of this long stripe is that it can minimize the interference from the connector during implantation. Then, an 8 mm×8 mm mesh structure is designed to further increase the device flexibility. On the top of this grid component, there is a 4×4 microneedle electrode array. The number of microneedle electrodes can be changed depending on the mask design. To communicate with the different layers in the brain to obtain various types of information, the length of these 3D microneedle electrodes also can be changed from 400 μm up to 3 mm via the design of the 2D mask pattern. As shown in Figure 1b, the hole size on the photomask determines the diameter of the drawing pillars. These drawing pillars are used to fabricate microneedle electrodes of specific lengths. The principle of this technology will be introduced in detail in the characterization section. Contrary to the approach proposed by Kwon *et al.*^29^, our fabrication technique can pattern the microneedle electrode array in a random profile. As mentioned in the literature, the array can have both a convex structure and a concave structure, which cannot be patterned by backside exposure from a photoresist droplet (Figure 1c).

### Fabrication process for the flexible microneedle electrodes

**Figure 2:**
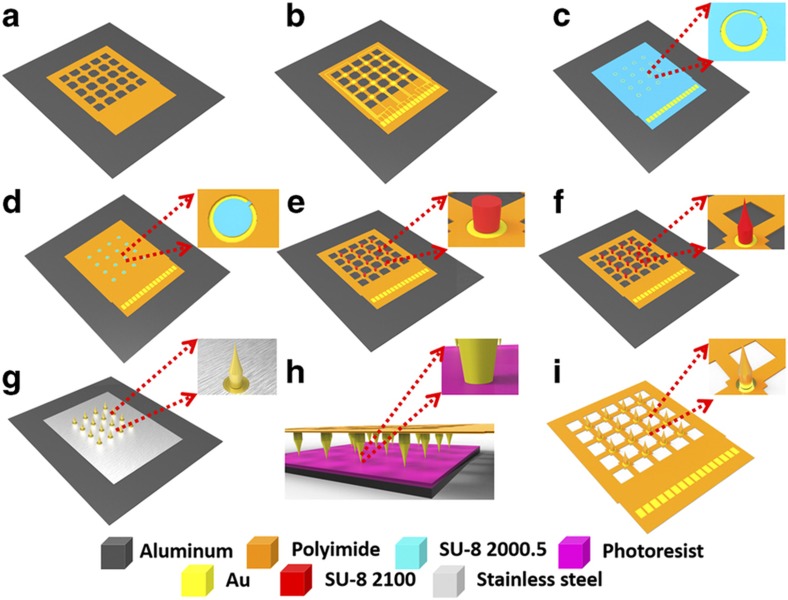
Fabrication process for the flexible microneedle electrode. (**a**) Bottom layer defined by UV lithography technology. (**b**) Metal tracing formed by lift-off process. (**c**) SU-8 adhesion layer patterning; (**d**) Top layer defined by UV lithography technology. (**e**) SU-8 pillar array formed by UV lithography technology. (**f**) SU-8 sharp tips formed by drawing lithography technology. (**g**) Gold layer deposition on the surface of microneedle electrode. (**h**) Parylene insulation layer deposition on the microneedle electrode. (**i**) Electrode release from the substrate.

The fabrication procedure followed standard photolithographic and clean room procedures. The process is shown in detail in Figure 2. First, a 1-μm-thick aluminium (Al) layer was evaporated onto a silicon substrate via physical vapor deposition. This layer acted as a sacrificial layer to release the final device from the substrate. Then, a 5-μm base layer of photosensitive polyimide (Durimide 7005, Fujifilm, Japan) was spun onto the Al-coated substrate. After being exposed to UV at a dosage of 120 mJ cm^−2^, the base layer was post baked and developed in HTRD2 and RER 600 (Fujifilm, Japan), which defined the bottom layer pattern of the mesh substrate (Figure 2a). The base polyimide layer was cured at 300 °C in N_2_ for 0.5 h. This baking process was designed to only partially evaporate the water in the polyimide layer. In this way, it would provide a chemically and physically stable surface for further processing while still leaving some unterminated bonds for attaching the top polyimide layer^30^. Then, a layer of 50 nm of Cr and 300 nm of Au was patterned on the top surface using a standard lift-off process. This defined the metal tracing and connecting pad on the bottom layer (Figure 2b). Next, a 0.5-μm layer of SU-8 2000.5 (Microchem, Westborough, MA, USA) was spun on the metal layer. This SU-8 layer was used as an adhesion layer for the SU-8 pillar structure (Figure 2c). The ring structure on the SU-8 layer was defined to ensure the metal connection between the bottom metal trace and the top sputtered metal layer on the microneedle electrode. An additional 5-μm-thick top layer of polyimide was spun onto the patterned metal layer and defined to expose the sensing contacts and connection pad (Figure 2d). The inset of Figure 2d demonstrates the concave openings of the contacts. Next, a 300-μm SU-8 2100 layer was deposited by two continuous spin-coating processes, which were patterned into the SU-8 pillar array (Figure 2e). The SU-8 sharp tips were formed using the drawing lithography technology we reported previously^31–33^ (Figure 2f). Drawing lithography technology is a maskless fabrication approach to build 3D structures based on the polymers’ different viscosities under different temperatures. In brief, a 200-μm-thick SU-8 layer was spun on a Si substrate and kept on a 95 °C hotplate to remove the solvent. A sample with developed SU-8 pillars was fixed on a 3D precision stage and aligned above the baked SU-8 layer. By adjusting the precision stage, the sample was lowered down until the SU-8 pillars were immersed in the baked SU-8. The baked SU-8 encapsulated the pillars’ surface due to its high viscosity. Then, the sample with SU-8 pillars was drawn away from the baked SU-8. During the drawing process, both the temperature and drawing speed were increased. Because the SU-8 is less viscous at higher temperature, the connection between the SU-8 pillars and surface of the baked SU-8 became individual SU-8 bridges, which shrank and then broke. The end of the shrunk SU-8 bridges formed sharp tips on the top of each SU-8 pillar when the connection separated. The SU-8 microneedle device was then UV exposed, post baked, hard baked and treated with oxygen plasma. This post-processing step not only induced a crosslink in the SU-8 to enhance the stiffness of the device but also removed the possible toxic leachants increasing its biocompatibility^34^.

**Figure 3:**
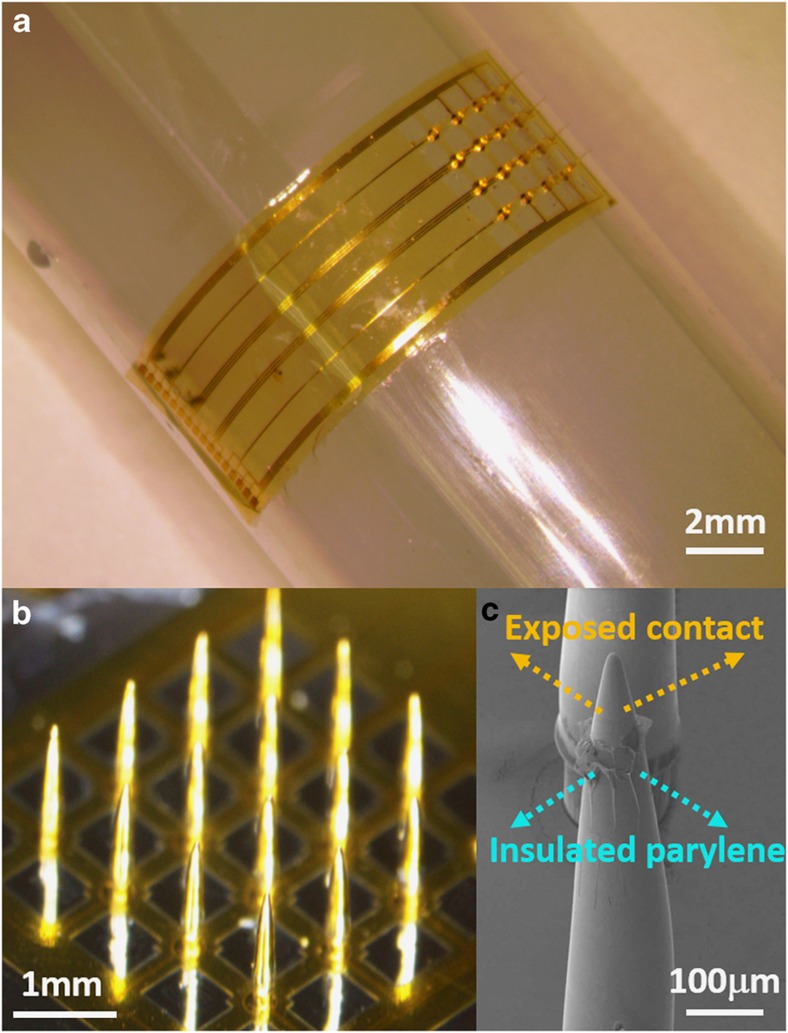
Fabrication result for a flexible microneedle electrode. (**a**) An optical image of the flexible microneedle electrode attached to a curved surface (scale bar: 2 mm). (**b**) Details of the microneedle electrode (scale bar: 1 mm). (**c**) SEM image of the exposed microneedle electrode contact (scale bar: 100 μm).

After the fabrication of the polymer SU-8 microneedle structure, a piece of stainless steel shadow mask was carefully aligned on the top of the sample. A gold layer was sputtered on the top of the polymeric microneedle for electrical conduction. This also enabled an electrical connection between the bottom metal tracing and the top microneedle electrode (Figure 2g). To improve the signal selectivity and spatial resolution of the metal-deposited microneedle electrode, a parylene-C insulation layer was deposited on top of the sputtered Au layer with openings only on the electrode tips (Figure 2h). The deposition of the insulation layer employed a special coating method reported by Byun *et al.*^35^. After a layer of viscous AZ 9260 was uniformly coated onto the silicon wafer, the sample was flipped over and lowered to dip the microneedle electrode tips into the photoresist. Then, the photoresist was dried on the hotplate at <65 °C, and the entire sample was loaded into the parylene coater for deposition. After the parylene-C was coated, the photoresist was removed using acetone and the microneedle electrode sample was separated. Finally, the entire device was released from the substrate via anodic metal dissolution of the Al sacrificial layer. Figure 3 shows the fabricated device. Due to the flexibility of the ultrathin polyimide mesh substrate, the entire device is conformal to a curved surface (Figure 3a). The microneedle electrodes on top were successfully developed (Figure 3b) and insulated with a parylene-C coating (Figure 3c).

## Results and discussion

### Length dependency of the microneedle electrodes

In conventional processes, the length of the microneedles is controlled by the baking temperature. The polymer’s viscosity decreases as the baking temperature increases, which leads to a lower extensional strain rate in the polymer. To a certain extent, the polymer’s gravity force is dominated by the extension force, and the drawn polymer’s ends shrink to sharp tips. However, the temperature distribution over the same sample is uniform, therefore it is not possible to pattern microneedle electrodes of different lengths on a single sample. Lee and Jung^36^ reported the relationship between the viscosity (*η*) and microneedle length *L*(*t*) in drawing lithography technology as:

(1)η(t)=FL0dL(t)dtπR2(t)−σdL(t)dtπR2(t)+O(Fi,Fg)

where *F* is the axial drawing force, *L*_0_ is the length at initial stage and *R*(*t*) is the radius of the drawn cylindrical liquid column. Because the surface tension force (second term of Equation (1)), initial force (*F*_*i*_) and gravitational force (*F*_*g*_) are ignored in this publication, the final equation is

(2)η(t)=FL0dL(t)dtπR2(t)

**Figure 4:**
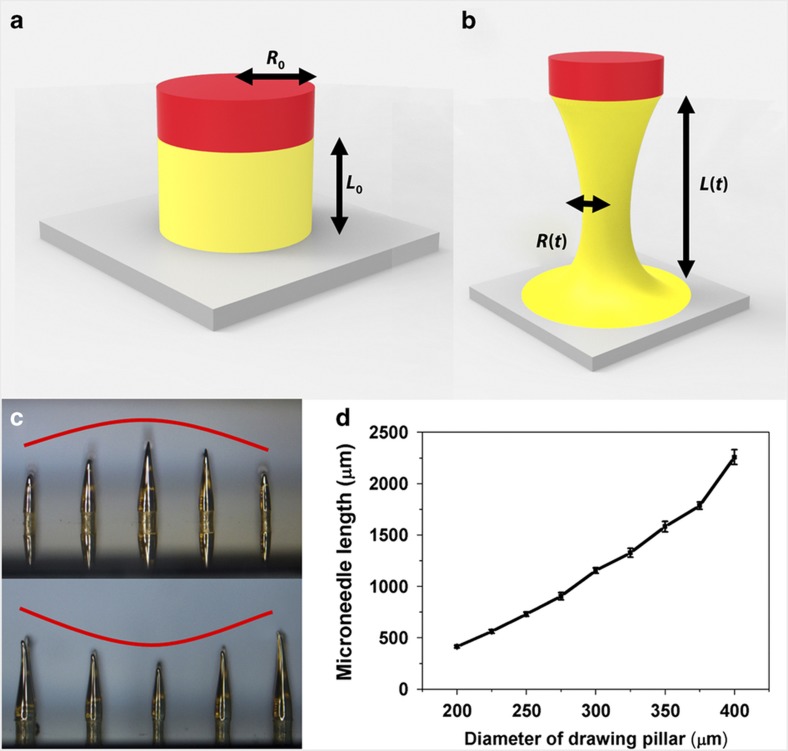
Fabrication of a microneedle array with different lengths. (**a** and **b**) The mathematic model for the drawing lithography process. (**c**) Convex and concave profiles of microneedle electrode arrays. (**d**) Microneedle electrodes with different lengths fabricated from different drawing pillars.

As shown in Figure 4, because the deformation was homogeneous and the drawn SU-8 was an incompressible fluid, the same volume is conserved in the entire process as

(3)R2(t)L(t)=R02L0

where *R*_0_ is the initial radius of the drawn cylindrical liquid column and

(4)R2(t)=R02L0L(t)

The axial necking region of the liquid bridge (radius shown as *R*(*t*)) is in steady state to maintain extensional deformation. However, the ultimate steady-state extensional viscosity is gradually decreased in a finite time; therefore, the initial necking radius of the liquid bridge will decrease. This capillary self-thinning of the unstable necking is not only affected by the liquid viscosity but also by the drawing speed. As the drawing speed increases, it takes less time for the liquid bridge to be drawn to the same length from the hotplate, where the heat dissipation is also smaller. This relative higher temperature will result in less viscosity, and the capillary self-thinning rate will increase, which will lead to a smaller radius *R*(*t*). However, in the experiment, all the drawing speeds are controlled at the same rate, and we only consider the differences resulting from the viscosity.

According to the Reynolds equation, the polymer viscosity can be expressed as

(5)η(t)=µ0e(−bT)

where *T* is the temperature, and *μ*_0_ and *b*_0_ are coefficients. Substituting Equation (4) and Equation (5) into Equation (2) results in the following equation:

(6)dL(t)L(t)dt=FπR02µ0e(−bT)

From Equation (6), it is obvious that the drawn length of the microneedle electrodes, *L*(*t*), primarily depends on the axial drawing force, *F*, the baking temperature, *T*, and the radius of the drawn cylindrical liquid column, *R*_0_. Because the drawn force, *F*, and baking temperature of the polymer, *T*, could be kept as constant throughout the entire process, the microneedle electrode length could be determined by the drawn cylindrical liquid column, *R*_0_, which was determined by the mask pattern for the SU-8 pillars. Therefore, we could pattern 3D slanted microneedle electrodes with different lengths in a random profile using just the 2D mask design for the SU-8 pillars.

Figure 4c shows microneedles with different lengths fabricated by changing the diameter of the SU-8 pillars rather than the control temperature or drawing speed as in the usual drawing lithography process. The fabricated device can be either a convex structure or a concave structure as designed in the 2D mask design. To study the relationship between the diameter of the SU-8 pillars on the sample and the corresponding length of the drawn microneedles, SU-8 pillars with different diameters were fabricated to draw microneedles. The varying diameters ranged from 200 μm to 400 μm with intervals of 25 μm. Ten samples were fabricated to calculate the errors in each diameter. The final results are shown in Figure 4d. The measurement data indicate that the microneedle electrodes fabricated using this approach were from 400 μm to 2.2 mm in length. The length of the microneedle electrode was controlled by the diameter of the SU-8 pillars. Using the arrangement of SU-8 pillars of different diameters on a mask, the fabricated sample can be patterned into any profile.

### Mechanical characterization of the fabricated microneedle electrode

**Figure 5:**
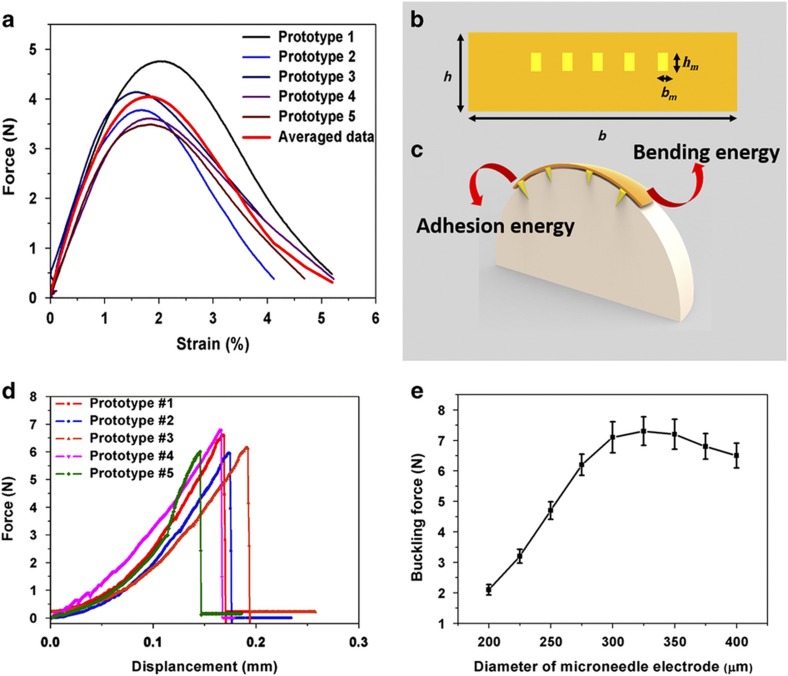
Mechanical characterization of the microneedle electrode. (**a**) Flexibility evaluation of the fabricated flexible microneedle electrode. (**b**) Cross section of the flexible substrate. (**c**) Schematic drawing of the attached flexible substrate on the curved brain surface. (**d**) The buckling tests of five different prototypes fabricated from a drawing pillar with a diameter of 300 μm. (**e**) The relationship between the buckling force and the diameter of the drawing pillars.

Because microneedle electrode arrays need to be applied to the curved surface of a brain, the main concern for a widely accepted device is whether it is conformal to the neural tissue. Therefore, the mechanical characteristics of the device were investigated. The tensile strength and elongation of five different prototypes were measured using the Instron Microtester 5848 (Instron, Norwood, MA, USA). The strain was obtained using a velocity of 10 mm min^−1^ up to the failure of the sample. The result is shown in Figure 5a. When the strain was <1.9%, the polyimide neural ribbon was in a reversible linear range, and the corresponding tensile strength was <4.03 N. When the strain was >1.9%, the neural ribbon was in an irreversibly deformed range, and the device formed a neck in its central region. The tensile strength decreased above the 1.9% strain range, and the device broke at greater elongation but lower stress. On the basis of the mathematic model given by Kim *et al.*^37^, the bending stiffness and bending energy can be obtained using the following equation:

$$EI=E_{PI}\mathrm{bh}\left(\frac{1}{3}h^{2}-hy_{0}+y_{0}^{2}\right)+\left(E_{Au}-E_{PI}\right)nh_{m}b_{m}\left[\frac{1}{3}h_{m}^{2}+h_{m}\left(h'-y_{0}\right)+{\left(h'-y_{0}\right)}^{2}\right]$$

$$\gamma_{b}=\frac{EI}{2R^{2}b}$$

where *E*_PI_ and *E*_Au_ are the Young’s Modulus of the polyimide and Au metal layers, respectively, the sizes *b*×*h and b*_*m*_×*h*_*m*_ are the dimensions of the polyimide layer and *n* gold bricks, respectively, *y*_0_ is the distance between the neutral axis and the bottom of the polyimide layer and *R* is the bending radius (Figure 5b). Because our fabricated flexible microneedle electrode was applied to a large brain surface area, the investigated bending radius was the radius of the cerebral hemisphere. On the basis of this mathematic model, the total bending energy of the device’s flexible substrate was only 0.89 mJ m^−2^, which is smaller than the reported adhesion energy for wet interfaces^38^. Therefore, after the microneedle electrode penetrates the brain, its flexible substrate can attach to the brain surface due to the surface tension effect of the adhesive body fluid. The deformed flexible substrate will be conformal to the curved brain and will not induce shear forces on the brain tissue via the inserted microneedles (Figure 5c).

Another common concern for the central neural interface is whether it is strong enough to penetrate the brain tissue^39^. To prove that the microneedles have sufficient stiffness for successful penetration, the buckling force was tested. Samples with a single microneedle electrode, which had varying diameters from 200 to 400 μm, were loaded under an axial compression using an Instron Microtester 5848 (Instron). Ten microneedle electrodes with the same length were tested in each group. The equipment drove the microneedle electrodes against a metal plate at a speed of 20 μm s^−1^ until each microneedle electrode broke. The failure loading point was observed when the loading sensor output had a sharp change. The force and its corresponding displacement data were recorded by a computer.

Figure 5d illustrates one representative example of buckling force testing for a microneedle electrode with a diameter of 300 μm. The axial force applied to the microneedle electrode increased with the plate displacement until the maximum load was reached. The fractured threshold was indicated by a discontinuity in the detected force and confirmed by visual observation during the test. After this fracture point, the loading plate continued to press against the crushed microneedle electrode with a decreased load. Figure 5e shows how the microneedles’ buckling force changed with their diameters. Initially, when the diameter was increased, the buckling force of the fabricated microneedle electrode also increased. However, when the diameter of the microneedle electrode was larger than 300 μm, its buckling force tended to be saturated. Further increasing the diameter decreased the buckling force. From Euler’s equation, $F=\frac{\pi^{2}EI}{{\left(KL\right)}^{2}}$, the buckling force is related to the area moment of inertia, *I*, and the microneedle electrode length, *L*. The area moment of inertia, *I*, is determined by the diameter of the microneedle electrode. Initially, the increase in the diameter leads the area moment of inertia to increase more than the length of microneedle electrode, and the buckling force became larger. When the diameter was larger than 300 μm, the increase in the microneedle electrode length was affected more than the growth of the area moment of inertia, which led to the buckling force saturating and then decreasing.

### *In vivo* measurements

The functionality of the fabricated flexible microneedle electrode was tested by implanting the device in a rat’s brain to record the spike signals. All procedures were approved by the Institutional Animal Care and the Use Committee at the National University of Singapore.

**Figure 6:**
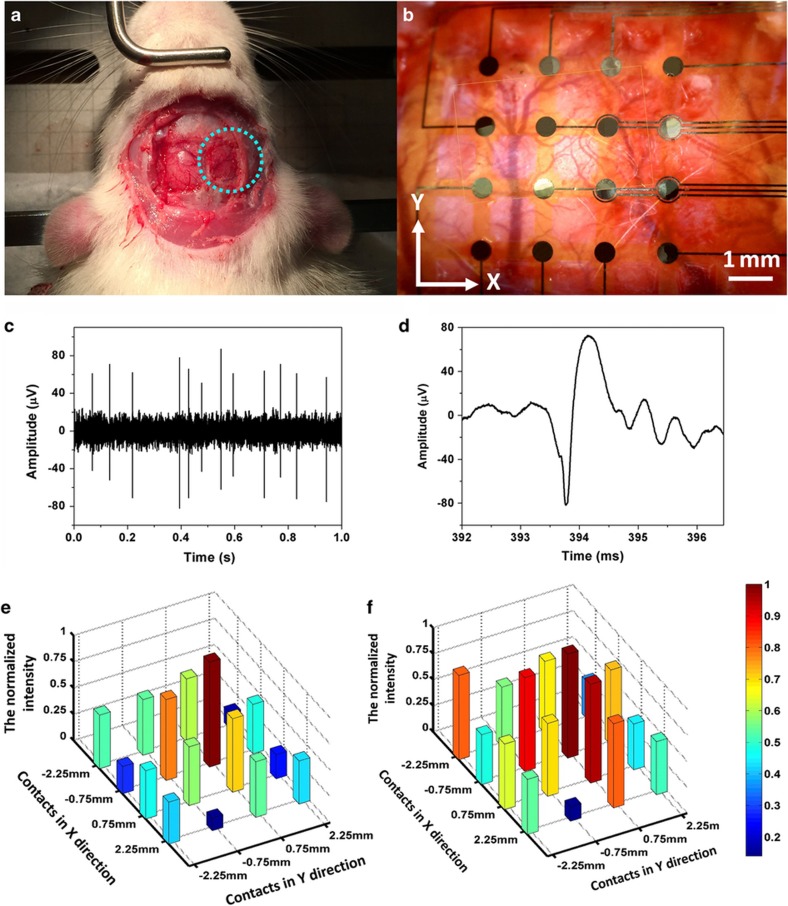
*In vivo* implantation of the fabricated flexible microneedle electrode. (**a**) Implanted location of the fabricated microneedle electrode array. The blue circle indicates the implantation location. (**b**) The mesh substrate is conformal to the curved brain surface after the implantation (scale bar: 1 mm). (**c**) A 1-s segment from the natural recordings of a representative electrode contact. (**d**) An enlarged portion of the signal. (**e**) The normalized natural neural activity from each electrode. The *x*–*y* axes indicate the location of each microneedle electrode. (**f**) The normalized neural activity after the stimulation is delivered to the hind paw.

Male Sprague-Dawley rats were anesthetized with ketamine hydrochloride (25 mg kg^−1^ intramuscularly) and surgical anesthesia was maintained with 2.5–5% Pentothal. The anesthesia level was monitored by continuous recordings of the heart rate, and the rectal temperature was maintained at 37–38 °C using a heating pad. The skull over the hemisphere was opened via craniotomy. The flexible microneedle electrode device was carefully mounted on a plate using a thin layer of maltose. After removing the dura, the flexible microneedle electrode and plate was inserted into the brain using a 3D micromanipulator system. Then, 0.5 ml of water was injected onto the plate to dissolve the maltose. Within several minutes, the maltose dissolved, and the plate was detached from the backside of the electrode. The flexible microneedle electrode device was left conformal to the brain surface (Figure 6b). A tungsten microwire was positioned on the cortical surface to be used as a reference electrode. All 16 microneedle electrodes were connected to an RZ5D BioAmp Processor (Tucker-Davis Technologies, Alachua, FL, USA) for signal acquisition (electrode impedance is shown in the Supplementary Information). (Figure 6c shows a 1-s egment from the natural recordings of a representative electrode contact. Of the 16 microneedle electrodes in 5 implantations, at least 7 out of 16 sites (43%) were successfully in recording neural activity. This proves that the spontaneous neural activity in the rat’s cortex was successfully recorded by the fabricated microneedle electrode.

Compared with the neural probe, one of the advantages of the microneedle electrode array is that it can simultaneously record neural signals from different locations in the brain. It is reported that these recordings can be correlated to the activity within clusters of locally connected cells to infer novel features of brain circuits^40^. After the rats were anesthetized, a 2-mA electrical stimulation was delivered through a stainless steel electrode to their hind paws. Local field potentials from the rat cortex were recorded by the fabricated microneedle electrodes to monitor the change in the neural activities. The responses of the evoked field potentials were quantitatively studied using the peak-to-peak value. The representative normalized recordings averaged over 10 min are shown in Figure 6. Each block represents the recorded amplitude from the electrode at a particular location. Compared with the natural neural activity (Figure 6e), recordings from each electrode increased after the stimulation was delivered through the hind paw (Figure 6f). In addition, a larger change appeared in the central part of the microneedle electrode array where the primary somatosensory cortex was located. These dynamic changes recorded by the microneedle electrode at each location may offer an approach to monitor travelling waves of local-field potential activity under external stimulations.

## Conclusions

This work successfully demonstrated that a 3D flexible microneedle electrode made of biocompatible materials could be fabricated using a new drawing lithography technology. The length of the microneedle electrode can be determined by the diameter of the drawing pillars. Using this technology, the length profile of the microneedle array can have an arbitrary profile, which cannot be realized by any other current methods. Using 2D designed patterns on a mask, the profile of the 3D microneedle electrode array can be controlled to access different functional layers in the brain. Due to its excellent flexibility and mesh structure, the deformed substrate is conformal to the curved brain and will not induce any shear force on the brain tissue via the inserted microneedles. The polymer-based microneedle electrode is strong enough to successfully penetrate the brain tissue and communicate with the neural system with its low impedance contacts. The *in vivo* tests on rats show that the flexible microneedle electrode can be implanted successfully into a brain with a curved surface and record neural signals. This flexible 3D electrode mesh offers a platform to integrate with other electronics to monitor chronic neural activity.

## References

[bib1] Chen C-H, Lin C-T, Hsu W-L et al. A flexible hydrophilic-modified graphene microprobe for neural and cardiac recording. Nanomedicine: Nanotechnology, Biology and Medicine 2013; 9: 600–604. 10.1016/j.nano.2012.12.004 23347893

[bib2] Chen C-H, Chuang S-C, Su H-C et al. A three-dimensional flexible microprobe array for neural recording assembled through electrostatic actuation. Lab on a Chip 2011; 11: 1647–1655. 21448485 10.1039/c0lc00718h

[bib3] Lin C-W, Lee Y-T, Chang C-W et al. Novel glass microprobe arrays for neural recording. Biosensors and Bioelectronics 2009; 25: 475–481. 19726175 10.1016/j.bios.2009.08.006

[bib4] Moxon KA, Leiser SC, Gerhardt GA et al. Ceramic-based multisite electrode arrays for chronic single-neuron recording. IEEE Transactions on Biomedical Engineering 2004; 51: 647–656. 15072219 10.1109/TBME.2003.821037

[bib5] Blanche TJ, Spacek MA, Hetke JF et al. Polytrodes: High-density silicon electrode arrays for large-scale multiunit recording. Journal of Neurophysiology 2005; 93: 2987–3000. 15548620 10.1152/jn.01023.2004

[bib6] Rui Y, Liu J, Wang Y et al. Parylene-based implantable Pt-black coated flexible 3-D hemispherical microelectrode arrays for improved neural interfaces. Microsystem Technologies 2011; 17: 437–442.

[bib7] Wang L-F, Liu J-Q, Yang B et al. PDMS-based low cost flexible dry electrode for long-term EEG measurement. IEEE Sensors Journal 2012; 12: 2898–2904.

[bib8] Bai Q, Wise KD, Anderson DJ. A high-yield microassembly structure for three-dimensional microelectrode arrays. IEEE Transactions on Biomedical Engineering 2000; 47: 281–289. 10743769 10.1109/10.827288

[bib9] Cheng M-Y, Je M, Tan KL et al. A low-profile three-dimensional neural probe array using a silicon lead transfer structure. Journal of Micromechanics and Microengineering 2013; 23: 95013.

[bib10] McAllister DV, Wang PM, Davis SP et al. Microfabricated needles for transdermal delivery of macromolecules and nanoparticles: Fabrication methods and transport studies. Proceedings of the National Academy of Sciences of the United States of America 2003; 100: 13755–13760. 14623977 10.1073/pnas.2331316100 PMC283494

[bib11] Gardeniers H. J. G. E., Luttge R, Berenschot EJW et al. Silicon micromachined hollow microneedles for transdermal liquid transport. Journal of Microelectromechanical Systems 2003; 12: 855–862.

[bib12] Wang R, Huang X, Liu G et al. Fabrication and characterization of a parylene-based three-dimensional microelectrode array for use in retinal prosthesis. Journal of Microelectromechanical Systems 2010; 19: 367–374.

[bib13] Wang R, Zhao W, Wang W et al. A flexible microneedle electrode array with solid silicon needles. Journal of Microelectromechanical Systems 2012; 21: 1084–1089.

[bib14] Kim K, Park DS, Lu HM et al. A tapered hollow metallic microneedle array using backside exposure of SU-8. Journal of Micromechanics and Microengineering 2004; 14: 597.

[bib15] Yung KL, Xu Y, Kang C et al. Sharp tipped plastic hollow microneedle array by microinjection moulding. Journal of Micromechanics and Microengineering 2012; 22: 015016.

[bib16] Yoon Y-K, Park J-H, Lee J-W et al. A thermal microjet system with tapered micronozzles fabricated by inclined UV lithography for transdermal drug delivery. Journal of Micromechanics and Microengineering 2011; 21: 025014.

[bib17] Moga KA, Bickford LR, Geil RD et al. Rapidly-dissolvable microneedle patches via a highly scalable and reproducible soft lithography approach. Advanced Materials 2013; 25: 5060–5066. 23893866 10.1002/adma.201300526 PMC4262250

[bib18] Wang P-C, Paik S-J, Chen S et al. Fabrication and characterization of polymer hollow microneedle array using UV lithography into micromolds. Journal of Microelectromechanical Systems 2013; 22: 1041–1053.

[bib19] Park J-H, Yoon Y-K, Choi S-O et al. Tapered conical polymer microneedles fabricated using an integrated lens technique for transdermal drug delivery. IEEE Transactions on Biomedical Engineering 2007; 54: 903–913. 17518288 10.1109/TBME.2006.889173

[bib20] Branner A, Stein RB, Fernandez E et al. Long-term stimulation and recording with a penetrating microelectrode array in cat sciatic nerve. IEEE Transactions on Biomedical Engineering 2004; 51: 146–157. 14723504 10.1109/TBME.2003.820321

[bib21] Vince V, Thil MA, Gérard AC et al. Cuff electrode implantation around the sciatic nerve is associated with an upregulation of TNF-alpha and TGF-beta 1. Journal of Neuroimmunology 2005;. 159: 75–86. 15652405 10.1016/j.jneuroim.2004.10.010

[bib22] Jeon M, Cho J, Kim YK et al. Partially flexible MEMS neural probe composed of polyimide and sucrose gel for reducing brain damage during and after implantation. Journal of Micromechanics and Microengineering 2014; 24: 25010.

[bib23] Yu H, Xiong W, Zhang H et al. A parylene self-locking cuff electrode for peripheral nerve stimulation and recording. Journal of Microelectromechanical Systems 2014; 23: 1025–1035.

[bib24] Fisher LE, Tyler DJ, Triolo RJ. Optimization of selective stimulation parameters for multi-contact electrodes. Journal of NeuroEngineering and Rehabilitation 2013;. 10: 25. 23442372 10.1186/1743-0003-10-25 PMC3599334

[bib25] Raspopovic S, Capogrosso M, Badia J et al. Experimental validation of a hybrid computational model for selective stimulation using transverse intrafascicular multichannel electrodes. IEEE Transactions on Neural Systems and Rehabilitation Engineering 2012; 20: 395–404. 22481834 10.1109/TNSRE.2012.2189021

[bib26] Lebedev MA, Nicolelis MAL. Brain-machine interfaces: Past, present and future. Trends in Neurosciences 2006; 29: 536–546. 16859758 10.1016/j.tins.2006.07.004

[bib27] Branner A, Stein RB, Normann RA. Selective stimulation of cat sciatic nerve using an array of varying-length microelectrodes. Journal of Neurophysiology 2001; 85: 1585–1594. 11287482 10.1152/jn.2001.85.4.1585

[bib28] Wark HAC, Sharma R, Mathews KS et al. A new high-density (25 electrodes/mm^2^) penetrating microelectrode array for recording and stimulating sub-millimeter neuroanatomical structures. Journal of Neural Engineering 2013; 10: 045003. 23723133 10.1088/1741-2560/10/4/045003

[bib29] Kwon KY, Weber A, Varying-Length WL. Polymer microneedle arrays fabricated by droplet backside exposure. Journal of Microelectromechanical Systems 2014; 23: 1272–1280.

[bib30] Lemmerhirt DF, Staudacher EM, Wise KD. A multitransducer microsystem for insect monitoring and control. IEEE Transactions on Biomedical Engineering 2006; 53: 2084–2091. 17019873 10.1109/TBME.2006.877115

[bib31] Xiang Z, Wang H, Pant A et al. Development of vertical SU-8 microtubes integrated with dissolvable tips for transdermal drug delivery. Biomicrofluidics 2013; 7: 026502. 10.1063/1.4798471 PMC3625238 24404018

[bib32] Xiang Z, Wang H, Pant A et al. Development of vertical SU-8 microneedles for transdermal drug delivery by double drawing lithography technology. Biomicrofluidics 2013; 7: 66501. 24396551 10.1063/1.4843475 PMC3869848

[bib33] Xiang Z, Wang H, Murugappan SK et al. Dense vertical SU-8 microneedles drawn from a heated mold with precisely controlled volume. Journal of Micromechanics and Microengineering 2015; 25: 025013.

[bib34] Vernekar VN, Cullen DK, Fogleman N et al. SU-8 2000 rendered cytocompatible for neuronal bioMEMS applications. Journal of Biomedical Materials Research 2009; 89: 138–151. 18431778 10.1002/jbm.a.31839

[bib35] Byun D, Cho SJ, Kim S. Fabrication of a flexible penetrating microelectrode array for use on curved surfaces of neural tissues. Journal of Micromechanics and Microengineering 2013; 23: 125010.

[bib36] Lee K, Jung H. Drawing lithography for microneedles: A review of fundamentals and biomedical applications. Biomaterials 2012; 33: 7309–7326. 22831855 10.1016/j.biomaterials.2012.06.065

[bib37] Kim D-H, Viventi J, Amsden JJ et al. Dissolvable films of silk fibroin for ultrathin conformal bio-integrated electronics. Nature Materials 2010; 9: 511–517. 20400953 10.1038/nmat2745 PMC3034223

[bib38] Chaudhury MK, Whitesides GM. Direct measurement of interfacial interactions between semispherical lenses and flat sheets of poly (dimethylsiloxane) and their chemical derivatives. Langmuir 1991; 7: 1013–1025.

[bib39] Xiang Z, Yen S-C, Xue N et al. Ultra-thin flexible polyimide neural probe embedded in a dissolvable maltose-coated microneedle. Journal of Micromechanics and Microengineering 2014; 24: 065015.

[bib40] Lewis CM, Bosman CA, Fries P. Recording of brain activity across spatial scales. Current Opinion in Neurobiology 2015; 32: 68–77. 25544724 10.1016/j.conb.2014.12.007

