# Supplementary material for: A flexible three-dimensional electrode mesh: An enabling technology for wireless brain–computer interface prostheses
Source: Microsyst Nanoeng. 2016 May 23;2:16012. doi: 10.1038/micronano.2016.12 (PMC6444742; doi:10.1038/micronano.2016.12)
Supplement: Supplementary Information [file micronano201612-s1.pdf]

Supplementary file

A flexible three-dimensional electrode mesh: An enabling technology for wireless brain–computer interface prostheses

Zhuolin Xiang[1,2,3,4], Jingquan Liu[5] and Chengkuo Lee[1,2,3,4]

Microsystems & Nanoengineering (2016) **2,** 16012; doi:10.1038/micronano.2016.12

IMPEDANCE OF FABRICATED MICRONEEDLE ELECTRODE

The electrode impedance of the microneedle electrode has been investigated to evaluate its electrical characteristics. Three-electrode setup was deployed to measure the electrochemical impedance spectroscopy versus a platinum reference electrode (Figure S1a). Phosphate buffered saline (PBS) with pH 7.4 at room temperature was used as the electrolyte for testing. A sinusoidal AC voltage with peak-to-peak amplitude of 10 mV at frequency from 0.5 Hz to 100 kHz was applied to measure the impedance of the microneedle electrode. The averaged measurement result from 25 single microneedle electrodes was shown in Figure S1b. The impedance of electrode contact showed a considerable dependence on frequency. At the biologically relevant frequency of 1 kHz, the impedance of the Au electrode was 847.6 kΩ. The standard equivalent circuit model for the electrode-electrolyte interface is also shown in Figure S1b. A constant phase element (for an ideal capacitor $n=1$) represented the interface between the electrode and saline. Rf was regarded as Faradaic impedance while Rs was taken as the spreading resistance of the solution. When the frequency increased from 0.5 Hz to 100 kHz, the non-frandic impedance, $Z_{dl}=1/(j\omega)^{n}C_{dl}$, decreased sharply. That is why the total impedance demonstrated an obvious dependence on the frequency and decreased at higher frequency ranges.

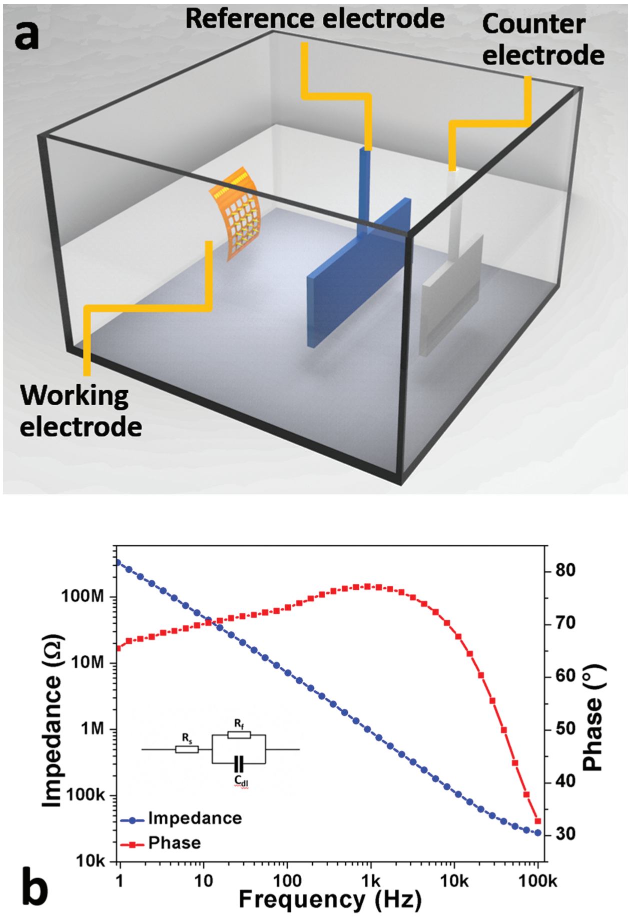

Figure S1 Impedance evaluation of the fabricated flexible micoroneedle electrode.

[1]Department of Electrical and Computer Engineering, National University of Singapore, 4 Engineering Drive 3, Singapore 117583, Singapore; [2]Singapore Institute for Neurotechnology (SINAPSE), National University of Singapore, 28 Medical Drive, #05-COR, Singapore 117456, Singapore; [3]Center for Intelligent Sensors and MEMS, National University of Singapore, 4 Engineering Drive 3, Singapore 117583, Singapore; [4]NUS Suzhou Research Institute (NUSRI), Suzhou Industrial Park, Suzhou 215123, China and [5]National Key Laboratory of Science and Technology on Micro/Nano Fabrication, Department of Micro/NanoElectronics, Shanghai Jiao Tong University, Shanghai 200240, China.
Correspondence: Chengkuo Lee (elelc@nus.edu.sg)
